# Distinct lung functional, histological and cell senescence signatures in the single and repetitive bleomycin mouse models of idiopathic pulmonary fibrosis

**DOI:** 10.14814/phy2.70560

**Published:** 2025-09-23

**Authors:** Jamal Bousamaki, Emma A. Rørbeck, Asbjørn Graver Petersen, Stefanie H. Korntner, Susanne E. Pors, Elizabeth F. Redente, Denise Oró, Casper Gravesen Salinas, Rebecca Wendelboe Olsen, Ulf Simonsen, Michael Feigh, Henrik H. Hansen

**Affiliations:** ^1^ Gubra A/S Hørsholm Denmark; ^2^ Department of Pediatrics National Jewish Health Denver Colorado USA; ^3^ Department of Biomedicine, Pulmonary and Cardiovascular Pharmacology, Faculty of Health Aarhus University Aarhus Denmark

**Keywords:** animal models, bleomycin, cellular senescence, disease phenotype, idiopathic pulmonary fibrosis, translatability

## Abstract

This study aimed to comprehensively compare the lung disease phenotypes between single‐dose and repetitive‐dose bleomycin (BLEO)‐induced mouse models of idiopathic pulmonary fibrosis (IPF). Male C57BL/6JRj mice were randomized and stratified to treatment according to body weight. Mice received either a single intratracheal instillation of BLEO (*n* = 14) or a repetitive regimen involving bi‐weekly BLEO instillations over 4 weeks (*n* = 30). Two weeks after the last BLEO dose, mice were assigned as baseline (*n* = 13) or repetitive BLEO‐IPF mice (terminated 8 weeks after baseline, *n* = 17). Saline‐treated mice served as healthy controls (*n* = 10 per model). The repetitive BLEO‐IPF mouse demonstrated sustained features of lung fibrosis, including persistent increases in lung hydroxyproline content, Ashcroft scores, and quantitative collagen levels 8 weeks after baseline. Histological analysis revealed ongoing pulmonary inflammation and accumulation of senescent myofibroblasts. Lung functional impairment was selective but persistent, with FEV0.1 being significantly reduced on study week 8. Lung transcriptome signatures in repetitive BLEO‐IPF mice were comparable to those reported in end‐stage IPF patients, albeit attenuated 8 weeks after baseline, suggesting initiation of reparative processes. The repetitive BLEO‐IPF mouse model recapitulates histological features of progressive lung fibrosis with an evolving cellular senescence phenotype, offering a relevant preclinical platform for studying IPF pathophysiology and evaluating long‐term effects of antifibrotic and senescence‐targeted therapies.

## 1 INTRODUCTION

Idiopathic pulmonary fibrosis (IPF) is a chronic and progressive interstitial lung disease characterized by the excessive deposition of extracellular matrix (ECM) components, including collagen and fibronectin, which results in tissue scarring and a gradual decline in lung function. The prognosis for IPF remains poor, with a median survival of 3–5 years following diagnosis (Leung et al., 2015; Martinez et al., 2017; Wolters et al., 2014). A major challenge in IPF research is the limited translatability of preclinical findings into clinical efficacy. Current animal models often fail to fully replicate the chronic and progressive features of human IPF, thereby hindering the development of novel therapeutic strategies (Maher & Wells, 2009; Moore et al., 2013).

The single (“acute”) intratracheal bleomycin (BLEO) instillation mouse model of IPF is widely used in preclinical IPF research and drug development (Li et al., 2022; Moeller et al., 2008). Pulmonary fibrosis is induced through BLEO‐mediated injury to alveolar epithelial cells (AECs), triggering a broad spectrum of pro‐fibrotic signaling, including cell‐cycle arrest, epithelial dysfunction, myofibroblast differentiation, and matrix remodeling (Cheresh et al., 2013; Della Latta et al., 2015; Heukels et al., 2019; Moore & Hogaboam, 2008). However, lung fibrotic injury in the single BLEO‐IPF mouse model resolves spontaneously over time (Jenkins et al., 2017; King et al., 2011), unlike human IPF, where fibrosis progresses over months to years and results in chronic, irreversible lung damage (King et al., 2011). Consequently, there remains a need for a murine model that more accurately reflects progressive and sustained fibrosis and epithelial remodeling seen in IPF^13^.

Repetitive BLEO instillations have recently been reported to induce more persistent (‘chronic’) features of pulmonary fibrosis (Degryse et al., 2010; Lv et al., 2022; Redente et al., 2021), more closely resembling the recurrent alveolar epithelial injury characteristic of IPF. As in IPF patients, this process is marked by sustained overactivation of fibroblasts and myofibroblasts, resulting in excessive and sustained collagen deposition and a consequent, progressive decline in lung function (Chung et al., 2003; Degryse et al., 2010; Leslie, 2012; Redente et al., 2021), enabling a broader time window for efficacy testing of anti‐fibrotic drugs. Interestingly, cellular senescence, a state of permanent cell cycle arrest induced by stress or damage, has recently emerged as an essential driver of fibrosis in IPF (Parimon et al., 2021; Schafer et al., 2017). Senescent cells are resistant to apoptosis, allowing them to persist and promote myofibroblast activation while inhibiting fibrosis resolution, thereby maintaining ECM deposition even in the absence of ongoing fibrogenesis (Lin & Xu, 2020; Martinez et al., 2017). Accumulation of senescent cells has been reported in the lungs of IPF patients, particularly in alveolar epithelial cell and fibroblast populations (Barnes et al., 2019; Parimon et al., 2021, 2023). Molecular constituents of the senescence‐associated secretory phenotype (SASP) has been proposed to be major mediators of the detrimental effects of senescent cells, involving synthesis and secretion of various signaling molecules, including cytokines, chemokines, growth factors, and proteases into the pulmonary tissue microenvironment (Parimon et al., 2021; Schafer et al., 2017), exhausting mechanisms of tissue repair to promote fibrosis. In accordance with clinical findings (Lin & Xu, 2020; Parimon et al., 2023), the repetitive BLEO‐IPF mouse model has been reported to exhibit significant upregulation of pulmonary SASP factors, including H2AX, transforming growth factor‐beta (TGF‐β), and various matrix metalloproteinases (MMPs), which can reinforce the fibrotic microenvironment (Degryse et al., 2010; Lv et al., 2022; Parimon et al., 2021; Schafer et al., 2017). The repetitive BLEO‐IPF mouse may therefore offer a more progressive and clinically relevant disease phenotype compared to the single BLEO‐IPF mouse, however, its applicability in preclinical IPF research remains to be fully validated. To address this gap, we systematically compared the lung disease phenotypes induced by repetitive versus single‐dose BLEO administration in mice, with a specific focus on features of lung fibrosis and cellular senescence.

## 2 MATERIALS AND METHODS

### 2.1 Ethics

All experiments were approved by the Danish Animal Experiments Inspectorate (license #2018‐15‐0201‐01532 and #2023‐15‐0201‐01454). All animal experiments followed Gubra bioethical guidelines, adhering to internationally accepted standards for the care and use of laboratory animals.

### 2.2 Animals

Male C57BL/6JRj mice were ordered from Janvier Labs (Le Genest Saint Isle, France) and housed in a controlled environment (12 h light/dark cycle, 21°C ± 2°C, humidity 50% ± 10%). Each animal was identified via an implantable subcutaneous microchip (PetID Microchip, E‐vet, Haderslev, Denmark). Mice had ad libitum access to tap water and chow (Altromin 1324; Brogaarden, Hoersholm, Denmark).

### 2.3 Single BLEO‐IPF mouse model

**FIGURE 1:**
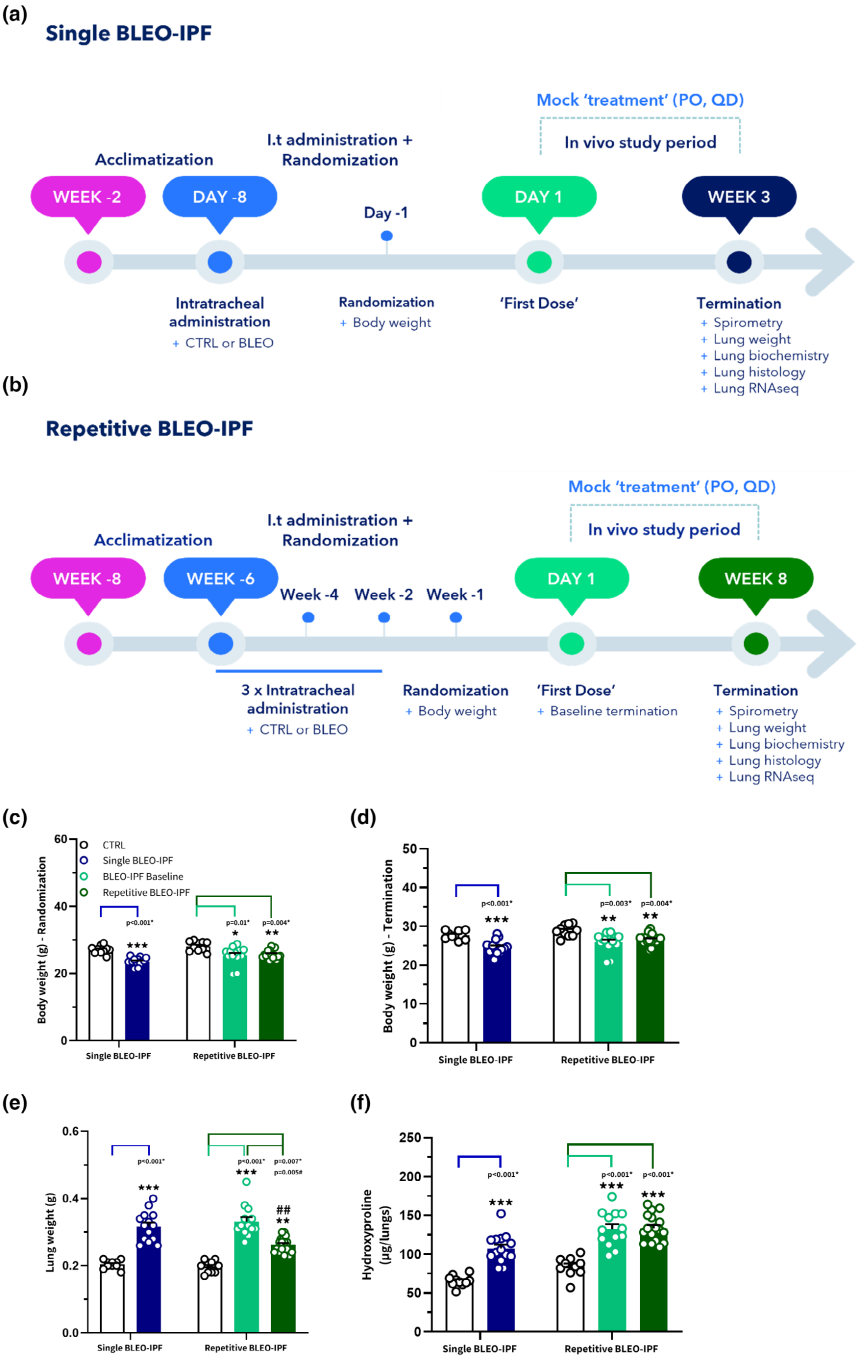
Study designs, metabolic and biochemical changes in the single vs repetitive BLEO‐IPF mouse model. (a) Single BLEO‐IPF mouse model study outline. Mice received a single intratracheal instillation of saline (CTRL) or bleomycin (BLEO‐IPF, 2.0 mg/kg, 3.0 U/kg) and were terminated 3 weeks after study start. (b) Repetitive BLEO‐IPF mouse model study outline. Mice received three intratracheal instillations of saline (CTRL) or bleomycin (BLEO‐IPF, 1.75 mg/kg, 2.63 U/kg) bi‐weekly over 4 weeks and were terminated on study day 1 (6 weeks after first BLEO dose, i.e. baseline) or study week 8 (14 weeks after the first BLEO dose). BLEO‐IPF mice in both studies were randomized and stratified to their respective groups according to (c) Body weight, measured 7 days after BLEO administration (single BLEO‐IPF), or 7 days after the last BLEO instillation (repetitive BLEO‐IPF). BLEO‐IPF mice were randomized and stratified to “mock” treatment based on body weight and were only considered eligible for inclusion if demonstrating a 5%–20% weight loss. (d) Terminal body weight. (e) Terminal lung weight. (f) Total lung hydroxyproline content. Data are presented as mean ± SEM. Single BLEO‐IPF mouse, ***p < 0.001 versus CTRL, unpaired *t*‐test. Repetitive BLEO‐IPF mouse, *p < 0.05, **p < 0.01, ***p < 0.001 versus CTRL; ^##^ *p* < 0.01 versus BLEO‐IPF baseline. Kruskal‐Wallis test with Dunn's multiple comparisons.

12‐week‐old male C57BL/6JRj mice received a single intratracheal instillation of bleomycin sulphate (cat. 0K071F; Baxter Healthcare, Deerfield; 2.0 mg/kg, 3 U/kg, dissolved in 50 μL sterile 0.9% saline, *n* = 12) or 0.9% saline as vehicle controls (50 μL) (Petersen et al., 2024) (Figure 1a for study outline). Saline‐instilled mice served as healthy controls, and all animals received vehicle peroral (PO), once a day (QD) for 3 weeks, to mimic handling during a treatment intervention study. Body weight was monitored daily, and animals were euthanized 21 days later.

### 2.4 Repetitive BLEO‐IPF mouse model

12‐week‐old male C57BL/6JRj mice received three intratracheal instillations of either BLEO (1.75 mg/kg, 2.63 U/kg, 50 μL) or vehicle (sterile 0.9% saline, 50 μL) bi‐weekly over 4 weeks (Figure 1b for study outline). BLEO animals were either assigned to the baseline group (*n* = 13, termination on study Day 1, i.e. 6 weeks after the first BLEO dose) or the repetitive BLEO‐IPF group (*n* = 17, termination at study week 8, i.e. 14 weeks after the first BLEO dose). Control mice receiving intratracheal instillations of saline (*n* = 10) were terminated at study week 8. All animals had body weight monitored daily and received vehicle (PO, QD) to mimic handling during an intervention study.

BLEO‐IPF mice were randomized and stratified to “mock” treatment based on body weight and were only considered eligible for inclusion if demonstrating a 5%–20% weight loss, a proxy for BLEO‐induced lung injury (Cowley et al., 2019), on day 7 after either single or last repetitive BLEO dose.

### 2.5 Pulmonary spirometry

Following terminal blood sampling, the animals were euthanized through cervical dislocation. Subsequently, a tracheostomy was performed, and an 18G (1 mm) metal cannula was inserted into the trachea, with the cannula secured using a suture. The animals were then connected to the flexiVent system (SCIREQ, Canada). To standardize lung volume and determine inspiratory capacity, a deep inflation was conducted, where the mouse lungs were inflated to a pressure of 30 cmH_2_O over 3 s, followed by a 3‐s pause at this pressure. Pressure‐volume (PV) loops were generated to assess the static compliance (Cst) of the respiratory system. A negative pressure‐driven forced expiratory maneuver was then executed by inflating the lungs to 30 cmH_2_O over 1 s, maintaining this pressure for 2 s, and subsequently connecting the animal's airways to a negative pressure reservoir (−50 cmH_2_O) for 2 s. Forced expired volume in 0.1 s (FEV0.1) and forced vital capacity (FVC) were directly calculated from the flow‐volume loops generated during lung deflation. Each maneuver was repeated in triplicate to ensure accuracy, with a coefficient of determination of 0.95 set as the threshold for accepting measurements.

### 2.6 Tissue sampling

Whole blood samples were collected in EDTA‐coated tubes and kept on ice. Blood samples were centrifuged for 10 min (4°C, 3000 *g*) to generate EDTA‐stabilized plasma. The supernatant was aliquoted and stored at −70°C for further analysis. The lungs were excised and weighed. Following this, the right lung was isolated by cutting the right bronchi, and samples were taken for biochemistry (superior and middle lobes) and RNA sequencing (inferior lobe). Right‐lung samples were stored at −70°C until processing, and the left lung was used for histology.

### 2.7 Lung hydroxyproline content

The right superior and middle lobe samples were homogenized in 6 M HCl and hydrolyzed to degrade collagen. The homogenate was centrifuged, and hydroxyproline (HP) content was measured in the supernatant using a colorimetric kit (Quickzyme Biosciences, Leiden, The Netherlands). Assessment of the whole‐lobe HP content in the right inferior, right superior, and left lung lobes after BLEO administration was performed in a previous study (Petersen et al., 2024).

### 2.8 Ashcroft fibrosis scoring and quantitative lung histology

The trachea of the isolated left lung lobe was cannulated, and perfusion fixed with 10% neutral‐buffered formalin (NBF) at a constant pressure for 5 min. Following this, the left lung was transferred to a vial containing 10% NBF and immersion fixed overnight at room temperature, transferred to 70% ethanol, and stored at 4°C until further processing. The tissue was then placed in a Tissue‐Tek VIP® 6 AI Vacuum Infiltration Processor (Sakura Finetek, Torrance, USA) to infiltrate prior to embedding. The lung was sectioned at a thickness of 4 μm using a microtome and mounted on Starfrost slides (Knittel, Braunschweig, Germany). For Ashcroft scoring, sections were stained with Masson's trichrome (MT, cat. B8586; Sigma‐Aldrich, Brøndby, Denmark). An automated deep learning‐based digital imaging analysis method [Gubra Histopathological Objective Scoring Technology, GHOST (Petersen et al., 2024)] was applied for automated histopathological scoring using the Ashcroft scoring system (Ashcroft et al., 1988; Hübner et al., 2008). For histomorphometric analysis, sections were stained with Picro Sirius red (PSR, cat. 365,548; Sigma‐Aldrich, Brøndby, Denmark), anti‐type I collagen (Col1a1, cat. 1310–01; Southern Biotech, Birmingham, AL), anti‐type III collagen (Col3, cat. 1330–01; Southern Biotech, Birmingham, AL), anti‐alpha‐smooth muscle actin (α‐SMA, cat. Ab124964; Abcam, Cambridge, UK), anti‐galectin‐3 (Gal‐3, cat. 125,402; Biolegend, San Diego, CA), anti‐CD45 (Abcam, cat. ab10558, Cambridge UK), anti‐CD3 (Abcam, cat. ab16669, Cambridge UK), anti‐CD20 (Santa Cruz Technologies, cat. sc‐7735, Dallas, Texas, US), or anti‐p21 (AbCam, cat. ab188224, Cambridge UK) using standard procedures (Petersen et al., 2024). Slides were scanned using a 20x objective (Aperio AT2, Leica Biosystems). Quantitative histology was performed using a digital imaging software (Visiomorph; Visiopharm, Hørsholm, Denmark) for the determination of whole‐section lung fibrosis (PSR, Col1a1, Col3), fibroblast activation (α‐SMA), inflammation (Gal‐3, CD45, CD20, CD3), and cell senescence (p21), respectively, expressed as positive staining relative (%) to total sectional area.

### 2.9 Histological analysis of myofibroblast senescence

4 μm sections were cut on a Leica rm2255 Rotary Microtome (Wetzlar, Germany) and stained with antibodies against α‐SMA (1:500; cat. A2547; Sigma Aldrich) and p16 (1:200; cat. ab211542; Abcam). Fluorescence‐conjugated secondary antibodies were used at 1:100 (goat anti‐mouse 555, cat. A‐21424; Donkey anti‐Rabbit 647, A‐31573; Thermo Fisher Scientific, Massachusetts, USA). Five images per mouse were captured and assessed in a blinded manner on a Zeiss Axiovert 200 M (Zeiss, Oberkochen, Germany) using a 20× objective and digital imaging software (AxioVision; Zeiss, Oberkochen, Germany) as previously described (Cooley et al., 2023). For each mouse, five representative fields of view were captured, and stereology grid counting was performed to quantify α‐SMA^+^ cells (indicating activated fibroblasts/myofibroblasts) and α‐SMA^+^/p16^+^ double‐positive cells (senescent myofibroblasts) by a single blinded observer. Per‐mouse averages were calculated from the twelve fields, and group‐level means were derived from the individual mouse averages within each experimental group.

### 2.10 Lung RNA sequencing

The inferior lobe samples were homogenized in lysis buffer, and RNA was purified using NucleoSpin® RNA binding strips (cat. no. 740698.5, Macherey‐Nagel, Dueren, Germany). RNA purity and concentration were measured using a NanoDrop 2000, and cDNA libraries were prepared using NEBNext® Ultra II Directional RNA library prep Kit for Illumina® (cat. no. E7760L, New England Biolabs, Ipswich, MA). cDNA libraries were sequenced to a depth of approximately 15 million reads per sample (single‐end, 75 bp reads) on a NextSeq 500 System (Illumina, San Diego, CA) using the NextSeq 500/550 High Output Kit version 2.5 (cat. no. 20024906, Illumina). Reads were aligned to the GRCm38 release 96 Ensembl *Mus musculus* genome using STAR version 2.7.0f (Dobin et al., 2013). All downstream analyses were performed using R version 3.6.0 (R Core Team, 2018). For differential gene expression analysis, the R package DESeq2 (Love et al., 2014) was used, and p‐values were corrected for multiple testing using the Benjamini‐Hochberg method (5% False Discovery Rate, FDR <0.05). Lung global gene expression changes in BLEO‐IPF mice were compared to previously reported RNA sequencing data from end‐stage IPF patients (Sivakumar et al., 2019). In addition, lung transcriptome signatures in BLEO‐IPF mice were probed against a curated set of 269 genes linked to human IPF, fibrosis and Senescence (Ma et al., 2022; McDonough et al., 2019; Roach et al., 2021; Sivakumar et al., 2019; Wollin et al., 2015; Zhao et al., 2022). Gene set enrichment analysis was conducted using the R package Piano (Love et al., 2014).

### 2.11 Statistics

Apart from deep learning‐based image analysis and RNA sequencing, data were analyzed using GraphPad Prism version 10.2.1 (GraphPad, La Jolla, CA). Results are presented as mean ± standard error of the mean (SEM). Normal distribution was assessed for both single and repetitive BLEO‐IPF datasets using the D'Agostino and Pearson omnibus normality test. For the single BLEO‐IPF study, normally distributed data were analyzed using an unpaired *t*‐test or Welch's *t*‐test (to account for unequal variances, if applicable). If normality was not met, data was analyzed using the Mann–Whitney *U* test. Power analysis was done with the R package pwr to determine minimum sample sizes based on empirical effect sizes. We confirmed >99% power to detect FVC differences ≥0.28 units (Cohen's *d* = 1.56) and >99.9% power to detect PSR collagen differences ≥7.2 units (*d* = 2.32), with minimum required sample sizes of 5 and 3 per group, respectively, for 80% power in the single BLEO‐IPF dataset. For the repetitive BLEO‐IPF dataset, normally distributed data were analyzed using a one‐way analysis of variance (ANOVA) followed by Dunnett's multiple comparisons test. If normality was not met, a Kruskal–Wallis test with Dunn's multiple comparisons test was applied. For datasets with unequal variances (assessed via Brown‐Forsythe test), Welch's one‐way ANOVA was used instead of standard ANOVA. A p value ≤0.05 was considered statistically significant. Power analysis showed >98% power to detect FVC differences (ANOVA *f* = 0.51) and >99% power for PSR comparisons (*f* = 0.68), with minimum sample sizes of 6 and 5 per group needed for 80% power, respectively.

To ensure robust detection of secondary endpoints and potential variability in fibrotic progression, sample sizes were increased for both study cohorts, exceeding minimum requirements. Statistical significance was set at *p* ≤ 0.05 (two‐tailed) for all analyses.

## 3 RESULTS

### 3.1 Comparable metabolic and biochemical profile in single versus repetitive BLEO‐IPF mouse model

Metabolic and biochemical profiles were assessed in two BLEO‐IPF mouse models established using either single‐or repeated BLEO instillation regimens (Figure 1a,b). In the single‐dose BLEO‐IPF mouse, the body weight of the BLEO‐IPF vehicle group was significantly reduced at randomization. It remained lower at termination, while lung weights and HP content were significantly increased compared to CTRL (Figure 1c–f). The baseline and 8‐week BLEO‐IPF groups from the repetitive BLEO‐IPF mouse showed a similar reduction in body weight at randomization and termination compared to the CTRL group (Figure 1c,d). Lung weights and HP content were also found to be increased in both BLEO‐IPF groups relative to CTRL, with lung weights being significantly lower at week 8 compared to baseline (Figure 1e,f).

### 3.2 Distinct lung functional deficits in single versus repetitive BLEO‐IPF mouse model

**FIGURE 2:**
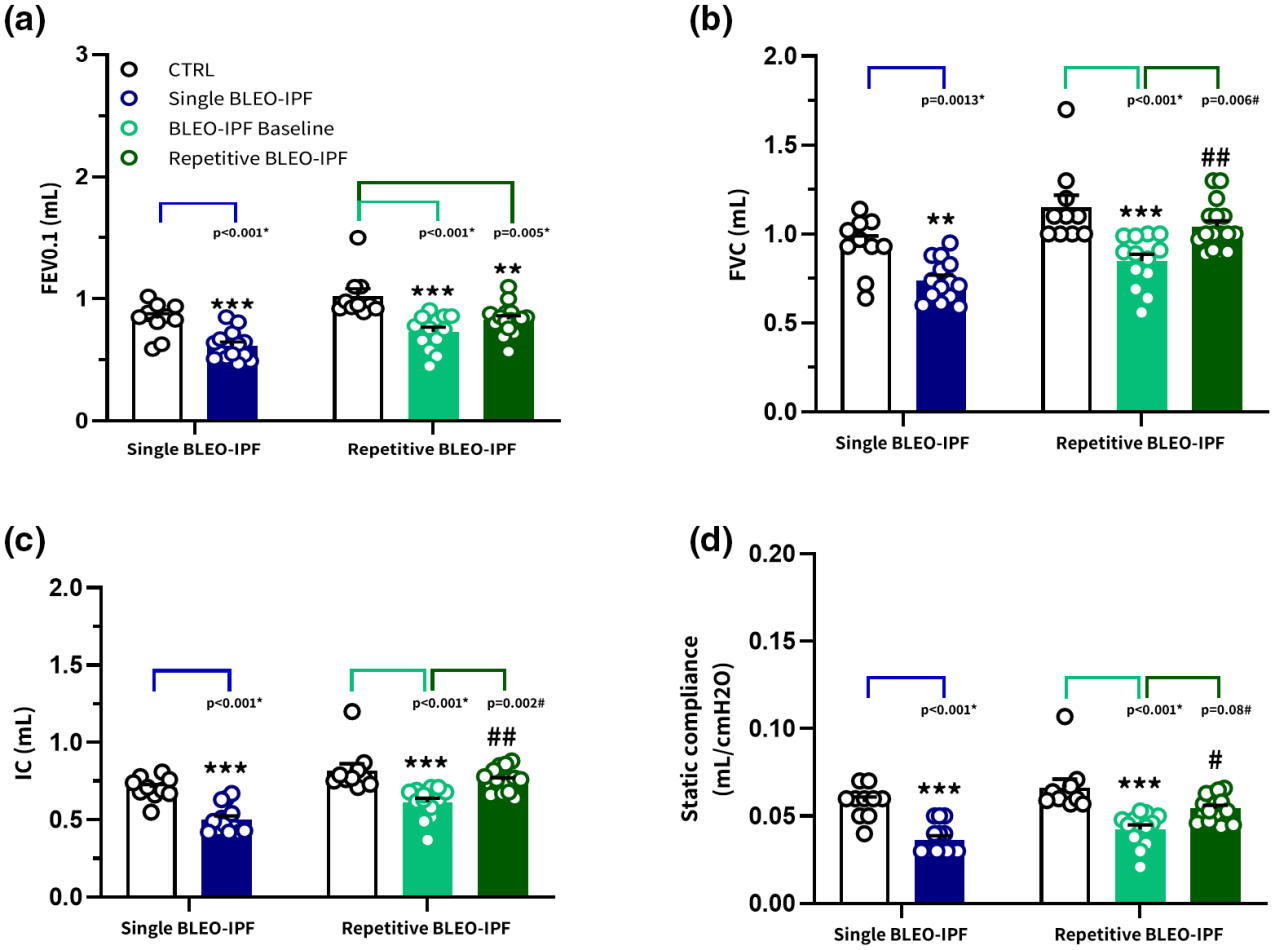
Spirometry profiling of the single versus repetitive BLEO‐IPF mouse model. Lung function assessment using spirometry. (a) Forced expiratory volume in 0.1 s (FEV0.1). (b) Forced vital capacity (FVC). (c) Inspiratory capacity (IC). (d) Static compliance. Data are presented as mean ± SEM. Single BLEO‐IPF mouse, **p < 0.01, ***p < 0.001 versus CTRL, unpaired *t*‐test. Repetitive BLEO‐IPF mouse, **p < 0.01, ***p < 0.001 versus CTRL; ^#^ *p* < 0.05, ^##^ *p* < 0.01 versus BLEO‐IPF baseline, Kruskal–Wallis test with Dunn's multiple comparisons.

In the single BLEO‐IPF mouse model, all assessed lung function parameters (FEV0.1, FVC, IC, Cst) were significantly reduced at the time of termination for the BLEO‐IPF vehicle group when compared with the corresponding CTRL group (Figure 2). Similar changes were found at baseline in the repetitive BLEO‐IPF mouse model, though this reduction was only retained for FEV0.1 8 weeks later (Figure 2a).

### 3.3 Histological evidence of persistent fibrosis in the repetitive BLEO‐IPF mouse model

**FIGURE 3:**
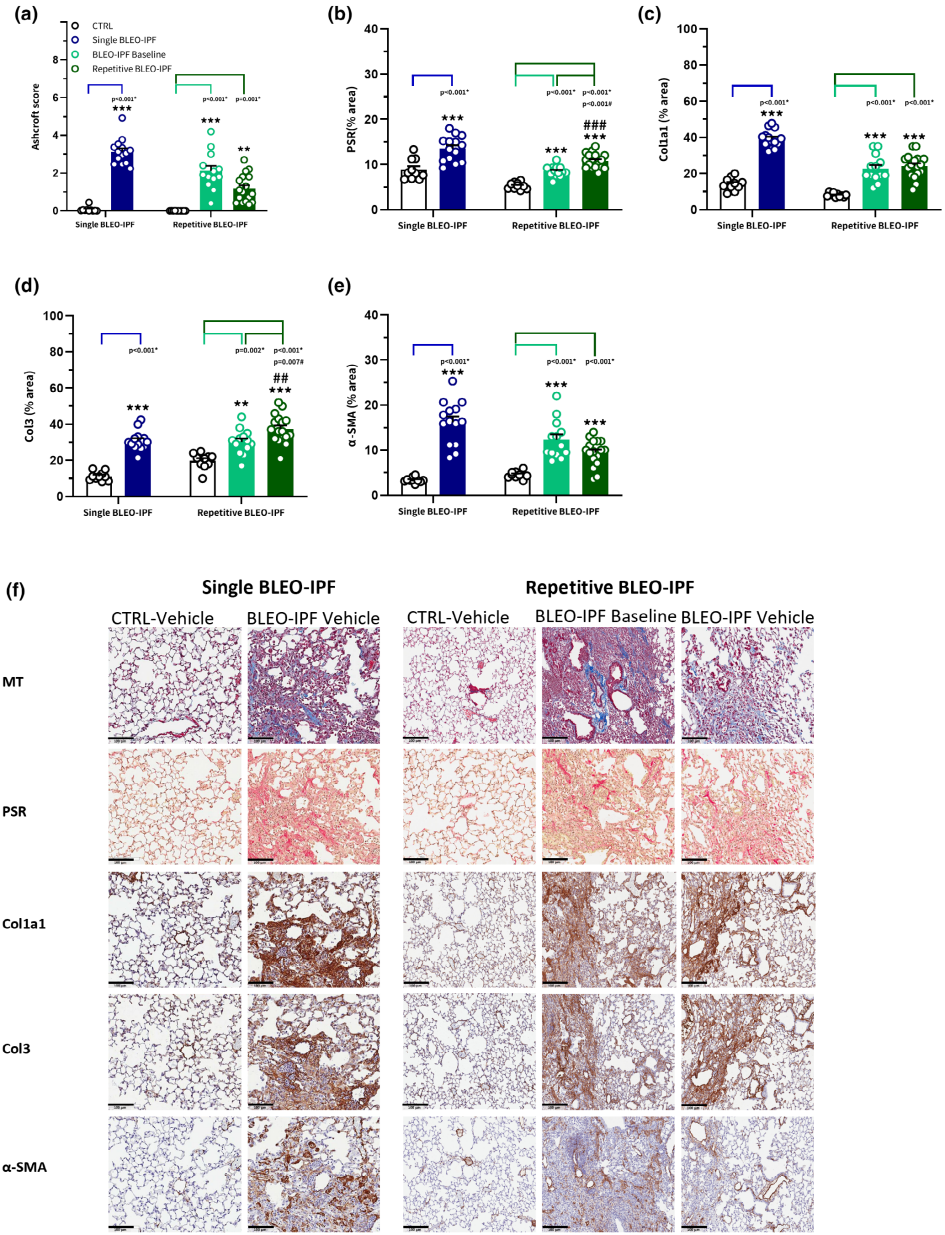
Histological features of pulmonary fibrosis in the single versus repetitive BLEO‐IPF mouse model. (a) AI‐based Ashcroft scoring. Histopathological scoring was performed on sections stained with Masson's trichrome (MT). Single BLEO‐IPF mouse, ***p < 0.001 versus CTRL, Mann–Whitney *U* test. Repetitive BLEO‐IPF mouse, **p < 0.01, ***p < 0.001 versus CTRL, Kruskal‐Wallis test with Dunn's multiple comparisons. (b–e) Histomorphometric assessment of fibrosis (PSR, Col1a1, Col3) and fibrogenesis (α‐SMA) using conventional image analysis. Data are expressed as proportionate (%) area of staining (mean ± SEM). (b) PSR. (c) Collagen‐1a1 (Col1a1). (d) Collagen‐3 (Col3). (e) α‐SMA. (f) Representative photomicrographs. Scale bar, 100 μm. Single BLEO‐IPF mouse, ***p < 0.001 versus CTRL, Welch's t‐test. Repetitive BLEO‐IPF mouse, **p < 0.01, ***p < 0.001 versus CTRL, ^##^ *p* < 0.01, ^###^ *p* < 0.001 versus baseline, Kruskal‐Wallis test with Dunn's multiple comparisons.

Lung fibrosis was evaluated using the AI‐based Gubra Histopathological Objective Scoring Technology (GHOST) platform (Figure 3a). Ashcroft score was significantly increased in the single BLEO‐IPF mouse as compared to CTRL. This finding was supported by quantitative histology (PSR, Col1a1, Col3, α‐SMA) (Figure 3b–e). In the repetitive BLEO‐IPF mouse, Ashcroft scores and quantitative histology markers (PSR, Col1a1, Col3, α‐SMA) remained elevated at both baseline and week 8. Notably, the fractional areas of PSR and Col3 was further increased at week 8 when compared to baseline measurements.

### 3.4 Distinct inflammatory profiles in the single versus repetitive BLEO‐IPF mouse model

**FIGURE 4:**
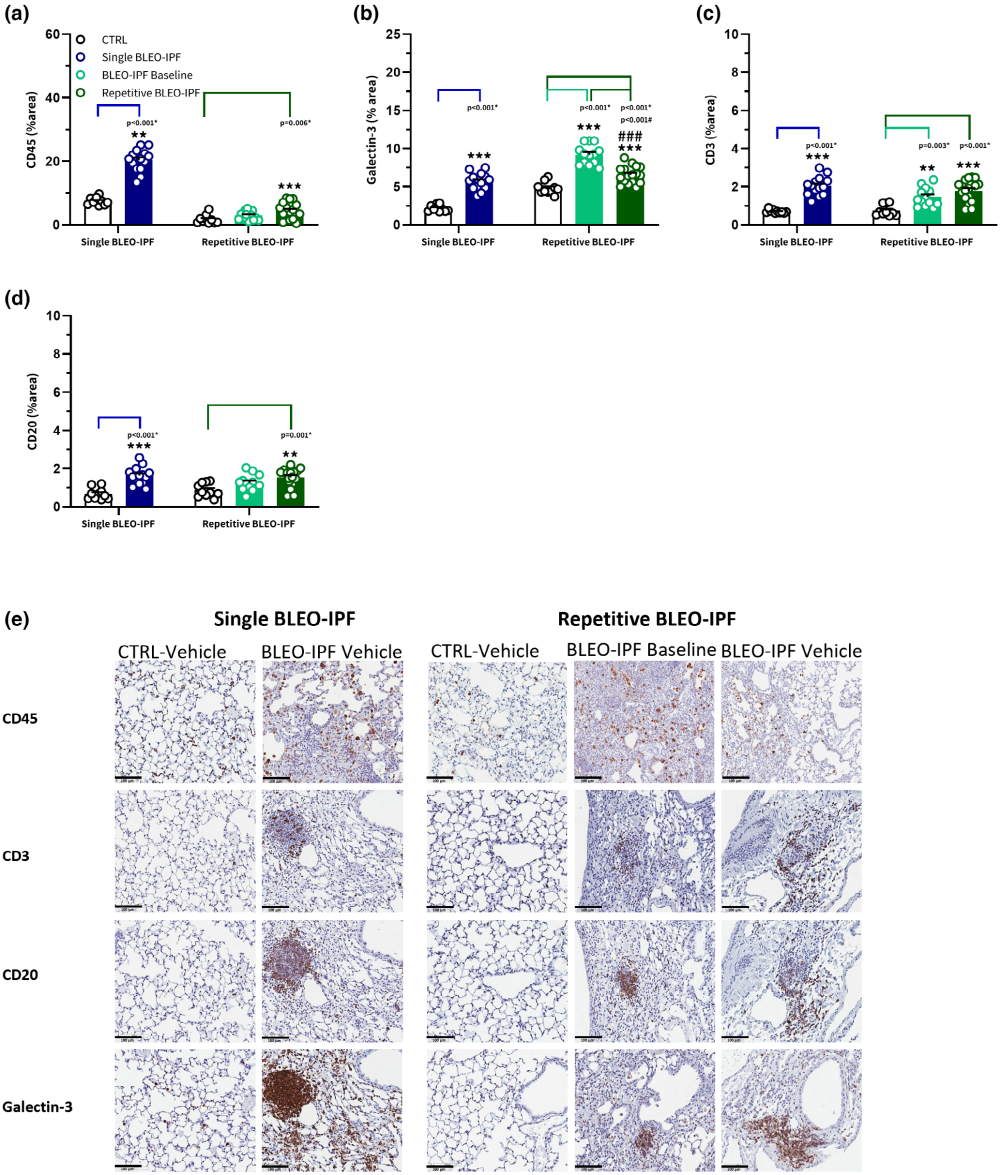
Histological features of pulmonary inflammation in the single versus repetitive BLEO‐IPF mouse model. Histomorphometric assessment of inflammation (CD45, Gal‐3, CD3, CD20) using conventional image analysis. Data are expressed as proportionate (%) area of staining (mean ± SEM). (a) CD45. (b) Gal‐3. (c) CD3. (d) CD20. (e) Representative photomicrographs. Scale bar, 100 μm. Single BLEO‐IPF mouse, **p < 0.01, ***p < 0.001 versus CTRL, Welch's t‐test (CD45, Gal3, CD3) or unpaired t‐test (CD20). Repetitive BLEO‐IPF mouse, **p < 0.01, ***p < 0.001 versus CTRL, ^###^ *p* < 0.001 versus baseline, Kruskal–Wallis test with Dunn's multiple comparisons (CD45) or oneway ANOVA with Dunnett's multiple comparisons (Gal3, CD3, CD20).

To evaluate inflammation in the two BLEO‐IPF mouse models, quantitative immunohistochemical markers of broad inflammatory cell populations, including CD45, Gal‐3, CD3, and CD20, were analyzed. The inflammation markers were chosen to assess immune populations known to be relevant in IPF pathogenesis, specifically CD45 (total leukocytes), CD3/CD20 (T‐ and B‐cell infiltration) (Cocconcelli et al., 2024; Heukels et al., 2019). Gal‐3 was prioritized over other macrophage markers due to its dual role in inflammation and fibrosis, with direct links to IPF progression (Bouffette et al., 2023; Hadjicharalambous & Lindsay, 2020). This combination captures innate (Gal‐3) and adaptive (CD3/CD20) immunity, avoiding redundancy with overlapping markers. In the single BLEO‐IPF model, BLEO‐IPF animals had significantly elevated levels of CD45, Gal‐3, CD3, and CD20 compared to CTRL (Figure 4a–d). In the repetitive model, Gal‐3 and CD3 levels were significantly elevated in both the baseline and 8‐week BLEO‐IPF groups compared to the CTRL group (Figure 4b,c). However, Gal‐3 levels were reduced at 8 weeks compared to baseline. In contrast, CD45 and CD20 levels were only found to be significantly elevated at week 8 (Figure 4a,d).

### 3.5 Distinct lung transcriptome signatures in the single versus repetitive BLEO‐IPF mouse model

**FIGURE 5:**
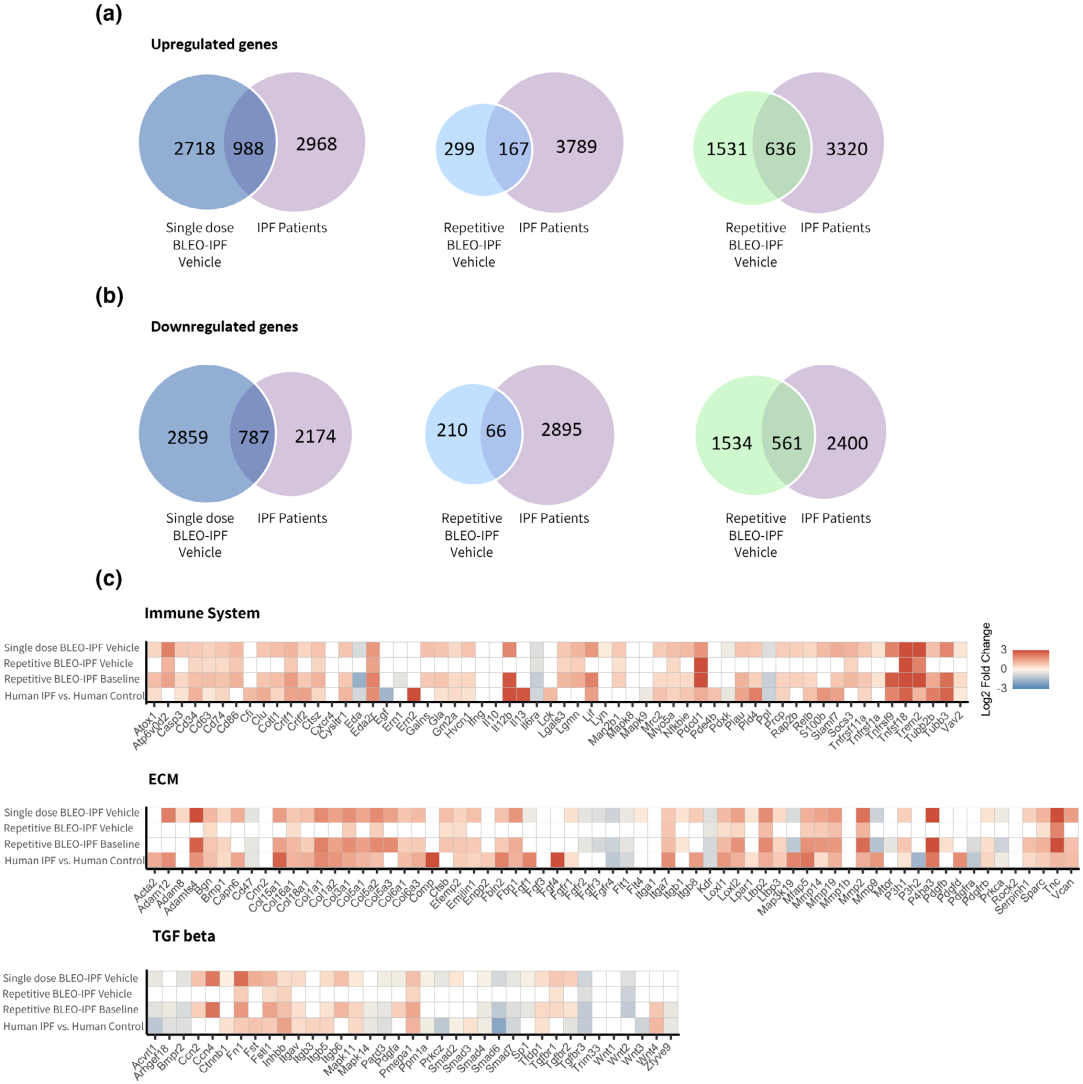
Lung transcriptome signatures. Lung transcriptome changes in single and repetitive BLEO‐IPF mice were validated against lung RNA sequencing data from IPF patients (Sivakumar et al., 2019). (a, b) Venn diagram depicting shared and separate differentially expressed genes (DEGs; false discovery rate *p* < 0.05) in single and repetitive BLEO‐IPF mice compared to IPF patients. (c) Curated list of 163 candidate genes linked to IPF pathogenesis and fibrosis, divided into three categories, i.e. extracellular matrix (ECM) organization, immune system and TGFβ‐associated signaling. Color gradients indicate significantly upregulated (red color) or downregulated (blue color) genes. White color indicates no significant change in gene expression compared to respective controls.

Lung gene expression changes associated with inflammation, ECM, and TGF‐β were analyzed for the single and repetitive BLEO‐IPF mouse models with reference to corresponding data reported in end‐stage IPF patients. Datasets were compared to the corresponding saline control group. Venn diagrams quantified DEG overlaps between mouse models and human IPF, while curated gene panels specifically compared transcriptional changes in inflammatory, ECM, and TGF‐β pathway genes (Figure 5a–c) (Sivakumar et al., 2019). The single BLEO‐IPF mouse exhibited the highest number of up‐ and downregulated DEGs when compared to CTRL (2718 and 2859 respectively), while also displaying transcriptional overlap with human IPF, capturing 25.0% (988/3956) of upregulated and 26.6% (787/2961) of downregulated DEGs. Moreover, the single BLEO‐IPF mouse displayed similar gene expression changes to the human IPF dataset, with marked upregulation of inflammatory mediators (*Il6, Ccl2*), ECM components (*Col1a1, Col3a1, Fn1, Loxl2, Mmp2*), and TGF‐β pathway signaling (*Tgfb1*, *Smad2*) (Figure 5c). In the repetitive BLEO‐IPF mouse, transcriptional alterations declined over the course of the study, with baseline samples showing 1531 and 1534 up‐ and downregulated DEGs, being reduced to 299 and 210 up‐ and downregulated DEGs by week 8 compared to respective controls. Transcriptional overlaps between the two models also decreased over time, with repetitive BLEO‐IPF animals capturing 16.1% (636/3956) and 19.0% (561/2961) of up‐ and downregulated DEGs at the baseline time point, declining to 4.2% (167/3956) and 2.2% (66/2961) 8 weeks later. This reduced number of DEGs at week 8 was paralleled by reduced expression of several fibrotic markers, although maintaining elevated expression compared to healthy controls. Inflammatory mediators (*Il6, Ccl2*), ECM components (*Col1a1, Col3a1, Fn1, Loxl2, Mmp2*) and TGF‐β signaling genes (*Tgfb1, Smad2*) all showed reduced expression compared to baseline but remained above control levels. This pattern was particularly evident for *Col1a1*, which showed near‐complete normalization, while *Loxl2* and *Col3a1* showed sustained elevations. While the single and repetitive BLEO‐IPF mouse models at baseline exhibited transcriptomic changes broadly similar directional changes in gene markers of pro‐inflammatory mediators, ECM components, and TGF‐β signaling, a number of DEGs in these pathways did not overlap between the BLEO models and human IPF. Whole ECM components, such as matrix metalloproteases showed broad cross‐species alignment (*Mmp14, Mmp19, Mmp2*); *Mmp9* was inversely regulated (upregulated in BLEO‐IPF mice versus downregulated in human IPF). Also, the regulation of fibroblast growth factor markers, such as *Fgf1* and *Fgf4*, as well as a subset of TGF‐β signaling markers showed more consistent upregulation in human IPF patients.

### 3.6 Distinct lung cellular senescence signatures in the single versus repetitive BLEO‐IPF mouse model

**FIGURE 6:**
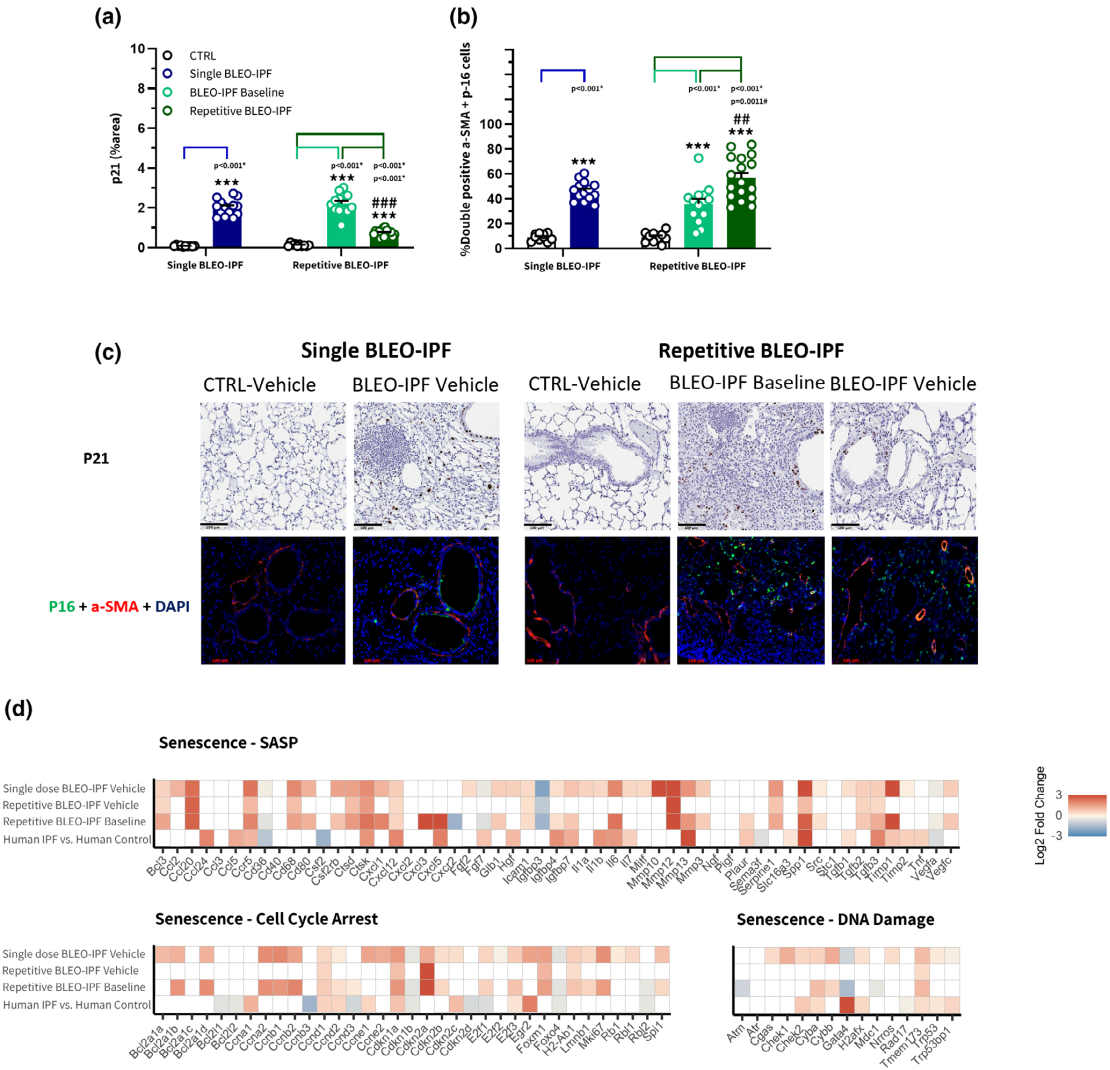
Histological and transcriptome signatures of pulmonary cellular senescence in the single versus repetitive BLEO‐IPF mouse model. (a) Quantitative histological analysis of p21 expression in the single and repetitive BLEO‐IPF mouse, using conventional image analysis. Data are expressed as proportionate (%) area of staining (mean ± SEM). (b) Quantification of double‐positive α‐SMA^+^/p16^+^ cells (senescent myofibroblasts). Data represent group means based on per‐mouse averages. (c) Representative photomicrographs. Scale bar, 100 μm. Single BLEO‐IPF mouse, ***p < 0.001 versus CTRL, Welch's *t* test. Repetitive BLEO‐IPF mouse, ***p < 0.001 versus CTRL, ^##^ *p* < 0.01, ^###^ *p* < 0.001 versus baseline, one‐way ANOVA with Dunnett's multiple comparisons. (d) Lung transcriptome signatures of cellular senescence in single and repetitive BLEO‐IPF mice were validated against lung RNA sequencing data from IPF patients (Sivakumar et al., 2019) cellular senescence, divided into three categories, i.e. Senescence‐Associated Secretory Phenotype (SASP), Cell Cycle Arrest and DNA Damage. Color gradients indicate significantly upregulated (red color) or downregulated (blue color) genes. White color indicates no significant change in gene expression compared to respective controls.

To gain insights on cellular senescence, immunostaining for p21, and co‐immunostaining for p16+/α‐SMA+ senescent myofibroblasts, along with the senescence‐associated transcriptomic signatures, was analyzed in the single and repetitive BLEO‐IPF mouse, respectively. For the single BLEO‐IPF mouse, there was a significantly elevated % area of p21, as well as a significant increase in the relative proportion (%) of double‐positive cells for p16 and α‐SMA compared to CTRL (Figure 6a,b). Transcriptomic data indicated a pronounced upregulation of p21 (*Cdkn1a*) and p16 (*Cdkn2a*), with p21 expression exceeding p16 (Figure 6d). The single BLEO‐IPF mouse mirrored the human IPF transcriptome profile, with induction of *H2afx* (DNA damage), p53 (*Trp53*), and *Il6*, alongside *Ccr5* (immune recruitment) and *Timp1* (ECM suppression) genes (Figure 6d). For the repetitive BLEO‐IPF mouse, p21 levels were elevated at baseline and 8 weeks versus CTRL but significantly lowered by week 8 compared to baseline (Figure 6a). Immunofluorescence revealed a significant increase in p16+/α‐SMA+ cells at week 8 relative to both baseline and CTRL (Figure 6b). RNA sequencing revealed that *Cdkn1a* expression was elevated at baseline but declined at week 8, while *Cdkn2a* was only increased at baseline (Figure 6d). Moreover, acute‐phase markers (*H2afx, Trp53, Il6, Timp1*) returned to CTRL levels by week 8 (Figure 6d).

## 4 DISCUSSION

We report that repetitive BLEO administration, followed by daily vehicle treatment to mimic handling during a preclinical therapeutic study, induces sustained histopathological features characteristic of IPF, along with distinct molecular signatures associated with pulmonary cellular senescence. Collectively, these findings highlight the repetitive BLEO‐IPF mouse as a relevant preclinical model in preclinical IPF research and may better enable long‐term intervention studies targeting pro‐fibrotic and cellular senescence‐associated mechanisms in IPF (Ma et al., 2022; Martinez et al., 2017; Moore & Hogaboam, 2008).

The current comparative study confirms and extends the findings of more persistent lung fibrotic injury in the repetitive versus single BLEO administration regimens in mice. As expected, histological markers of fibrosis were upregulated 4 weeks after single BLEO instillation. It is important to note that a key limitation of this model is the consistent, spontaneous regression of pulmonary fibrosis, which typically begins around 3 to 4 weeks post‐administration, as previously reported by our group and others (Della Latta et al., 2015; Izbicki et al., 2002; Peng et al., 2013; Petersen et al., 2024). In contrast, the repetitive BLEO‐IPF mouse demonstrated elevated lung HP content along with sustained fibrotic injury assessed by Ashcroft scoring and quantitative histology (PSR, Col1a1, Col3) over the entire study duration of 14 weeks. Increased pro‐fibrotic activity was reflected by enhanced α‐SMA expression, a standard marker of myofibroblast accumulation (Holm Nielsen et al., 2019). Notably, PSR staining and Col3 immunostaining were significantly elevated relative to baseline. In comparison, Col1a1 immunostaining appeared stabilized at this time point. As PSR predominantly labels type I and III collagens (López De Padilla et al., 2021), this is likely indicative of advancing deposition of particularly Col3‐containing fibers in the repetitive BLEO‐IPF mouse. Hence, the findings of the present study underscore that the fibrotic features observed in the repetitive BLEO‐IPF mouse are distinct from the histopathological phenotype in the corresponding single‐dose model (Yanagihara et al., 2020). A study characterizing a similar repetitive BLEO instillation regimen in mice, using a lower BLEO dose (1 U/kg), also reported progressive increases in lung collagen content at 28 weeks after the first instillation, suggesting that lowering the BLEO dose results in a slower rate of fibrotic lung injury, while we observe signs of progressive fibrosis already 14 weeks after the first instillation. This could likely be caused by the higher BLEO dose (2.63 U/kg) used in our model, indicating that increased BLEO exposure may accelerate fibrotic remodeling in the repetitive BLEO‐IPF mouse model. The single BLEO‐IPF mouse demonstrated more consistent upregulation of a wide selection of gene expression markers of fibrosis. It should be noted that transcriptomic signatures indicative of enhanced collagen synthesis in the single BLEO‐IPF mouse are transient, gradually resolving toward normalization approximately 6 weeks post‐dosing (Peng et al., 2013; Petersen et al., 2024; Selman & Pardo, 2021). While our lung RNA sequencing analysis corroborated histological findings of fibrosis in the repetitive BLEO‐IPF mouse at the baseline time point, a subset of structural ECM components (*Col1a1, Col3a1, Fn1, Loxl2, Mmp2*) and TGF‐β signaling (*Tgfb1, Smad2*) were attenuated or resolved at the study week 8 time point. This transition in ECM remodeling at week 8 is indicative of highly complex dynamics in the regulation of collagen turnover in the model, which aligns with previous studies' findings (Bonatti et al., 2023; Chung et al., 2003). Collectively, these temporal dynamics most likely reflect ongoing ECM remodeling and tissue repair mechanisms in response to BLEO toxicity, involving the contribution of a wide array of molecular mechanisms regulating fibrogenesis and collagen degradation (Budi et al., 2021; Selman & Pardo, 2021).

Furthermore, while both BLEO‐IPF models broadly mirrored the transcriptome signature of human IPF, divergences across a number of fibrosis signaling markers were noted. Functionally, these differences indicate that while the repetitive BLEO‐IPF model exhibits a broad pro‐fibrotic transcriptome signature, it does so in a temporally restricted and injury‐driven manner that does not reproduce the persistent growth factor and TGF‐β signaling observed in end‐stage human IPF. (MacKenzie et al., 2015; Sivakumar et al., 2019) The progressive decline in DEG overlaps between the two BLEO‐IPF models indicates that each model captures a subset of the highly complex lung transcriptome changes in human IPF, consistent with the repetitive BLEO‐IPF model representing a relatively stable lung fibrotic state (Degryse et al., 2010; Redente et al., 2021). Moreover, these transcriptional differences illustrate that preclinical models of IPF and human IPF represent distinct pathophysiological states, which can be considered complementary rather than conflicting in the context of preclinical research (Jenkins et al., 2017; Moore et al., 2013; Sundar & Matson, 2024).

In addition to lung fibrotic characteristics, the repetitive BLEO‐IPF mouse exhibited histological features of persistent inflammation, as indicated by increased staining of CD45 (pan‐leukocyte marker), CD20 (B‐cell marker), and CD3 (T‐cell marker) measured at study week 8. Sustained CD20+ B‐cell presence has been linked to immune cell aggregation in non‐lymphoid tissues, referred to as tertiary lymphoid structures (TLS), in IPF patients (Cocconcelli et al., 2024). TLS formation correlates with accelerated disease progression and lung functional decline in IPF (Cocconcelli et al., 2024; Heukels et al., 2019). Supporting this, B‐cell‐derived cytokine release has been shown to promote fibroblast migration and proliferation in IPF patients (Ali et al., 2021). Therefore, the elevated CD20 signal in the repetitive BLEO‐IPF mouse may reflect immune mechanisms perpetuating fibrosis, potentially through sustained B‐cell activation. In contrast, Gal‐3, a marker of activated macrophages (Bouffette et al., 2023), was significantly reduced in the repetitive BLEO‐IPF mouse at 8 weeks compared to baseline, yet remained significantly elevated relative to healthy control mice, suggesting a gradual resolution of macrophage infiltration as previously reported in comparable BLEO models (Degryse et al., 2010; Redente et al., 2021).

Contrasting histological findings of persistent lung fibrosis, the repetitive BLEO‐IPF mouse exhibited transient lung functional impairment, with only FEV0.1 being significantly reduced at 8 weeks compared to baseline, and static compliance (lung elasticity) showing borderline significance (*p* = 0.08) toward impairment compared to CTRL. Overall, the spirometry analysis suggests early airflow limitation during rapid expiration, suggesting sustained mild lung functional impairment in the repetitive BLEO‐IPF mouse. In comparison, the single BLEO‐IPF mouse showed broad declines across all measured spirometry endpoints (FVC, FEV0.1, IC, and Cst) at 4 weeks post‐BLEO. Importantly, however, FEV0.1, Cst, and FVC have consistently been reported to normalize around 6 weeks post‐BLEO in the single‐BLEO mouse (Della Latta et al., 2015; Petersen et al., 2024; Redente et al., 2014). It should be emphasized that this study is the first to report a complete lung functional profile using spirometry in a repetitive BLEO‐induced IPF model in mice. To date, spirometry data reported in comparable repetitive BLEO‐IPF models are limited to static compliance (Lv et al., 2022; Redente et al., 2021). Also, mouse models of silica‐induced lung fibrosis have been reported to show persistent fibrosis without concurrent changes in spirometry results (Dekoster et al., 2020; Janssen et al., 2024), indicating that a disconnect between lung histological and functional outcomes may occur in these complex models, and further comprehensive studies are needed. Several factors may explain the disparity in lung function between the repetitive and single BLEO‐IPF mouse models. Early small‐airway remodeling and the substantial airway reserve capacity of rodents may mask lung functional deficits (Irvin & Bates, 2003; Peng et al., 2013; Polosukhin et al., 2012; Verleden et al., 2020). The disparity could also reflect natural recovery, similar to the spontaneous resolution seen in the single BLEO‐IPF model (Moeller et al., 2008; Redente et al., 2023). In this context, resolution of inflammation and pulmonary oedema in the repetitive BLEO‐IPF model may alleviate functional deficits, as suggested for the single‐dose BLEO model (Petersen et al., 2024). Nevertheless, extended longitudinal monitoring might reveal greater ECM remodeling and more consistent lung functional impairment as reparative mechanisms become exhausted.

Pulmonary cellular senescence is increasingly recognized as playing a key role in the onset and progression of IPF. Senescent cells, induced by stressors such as telomere attrition or oxidative damage, adopt a SASP that drives inflammation and ECM remodeling, thereby contributing to the persistence of lung fibrosis in IPF (Barnes, 2019; Chin et al., 2023; Hernandez‐Segura et al., 2018). To gain further insight into the role of cellular senescence in BLEO‐based models of IPF, we assessed histological and transcriptional markers of cellular senescence after repetitive and single intratracheal BLEO instillation. In both models, immunohistochemical analyses revealed persistently elevated levels of the standard cellular senescence markers p16 and p21 (Lei et al., 2024; Lv et al., 2022). In the repetitive BLEO‐IPF mouse, further increases were observed for the number of p16/α‐SMA double‐positive myofibroblasts compared to baseline. In contrast, p21 levels declined over the course of the study in the repetitive BLEO‐IPF mouse. This divergence could potentially reflect myofibroblast heterogeneity and/or distinct roles of p16 and p21 in cellular senescence signaling. Accordingly, expression of p21 (p53‐driven early‐response inhibitor) declines as acute DNA‐damage signaling resolves, whereas p16 enforces irreversible cell‐cycle exit, leading to sustained upregulation of apoptosis‐resistant senescent myofibroblast subpopulations (Barnes, 2019; McHugh et al., 2024; Parimon et al., 2021). Our findings are supported by earlier studies in comparable model settings, which report the accumulation of senescent myofibroblast markers (Degryse et al., 2010; Lv et al., 2022). The single BLEO‐IPF mouse more closely mirrored findings of pulmonary cell senescence in end‐stage human IPF, featuring significantly increased expression of various senescence markers, including *Cdkn1a, Cdkn2c, H2afx, Trp53, Il6, Ccr5*, and *Timp1*. These SASP‐associated factors are known to trigger fibrosis via persistent DNA damage, immune cell recruitment, and ECM stabilization (Hernandez‐Segura et al., 2018; Russo & Ryffel, 2024; Selman et al., 2000). For the repetitive BLEO‐IPF mouse, these responses were mainly attenuated 8 weeks after baseline. Collectively, the different transcriptional dynamics in the repetitive BLEO‐IPF mouse suggest that senescent cells contribute differentially to sustain lung fibrosis, i.e. p21/p53‐dependent DNA damage responses play a significant role in the initiation phase, followed by a transition to p16‐driven maintenance of fibrosis (Janssen et al., 2024; Verleden et al., 2020).

Given the histological features of sustained fibrotic injury, the repetitive BLEO‐IPF mouse model may enable more reliable assessment of anti‐fibrotic drug efficacy by offering a significantly longer treatment window. Moreover, preservation of IPF‐associated pathology in the repetitive BLEO‐IPF mouse may allow the identification of molecular mechanisms relevant to preclinical drug discovery for IPF. It is worth noting that our spirometric and transcriptome analyses revealed partial regression of lung disease in the repetitive BLEO‐IPF mouse model. Hence, developing multiple‐hit models by combining repetitive BLEO with an additional pro‐fibrotic insult induced by intratracheal administration of lipopolysaccharide (LPS), Adeno‐associated virus (AAV)‐mediated pulmonary overexpression of TGFβ1 or repetitive BLEO administration in transgenic mouse models of IPF (Kim et al., 2022; Kurniawan et al., 2023; Lawson et al., 2005; Strobel et al., 2022), may potentially offer models with enhanced features of progressive fibrosis and thus improved clinical translatability. Limitations of our study should be acknowledged. We did not include an additional group to control for phenotype characteristics at 8 weeks after single BLEO administration, thereby limiting direct comparisons of the two models across time points assessed in the study. Also, follow‐up analyses beyond 14 weeks post‐dosing would be advantageous to delineate further features of the chronicity of fibrotic injury upon repetitive BLEO instillations.

## 5 CONCLUSION

Our study highlights the repetitive BLEO‐IPF mouse as a relevant platform in preclinical research and drug development for IPF. Importantly, the repetitive BLEO‐IPF mouse model shows histological features of persistent lung fibrosis, thereby enabling a longer treatment intervention window compared to the single BLEO‐IPF mouse.

## AUTHOR CONTRIBUTIONS

AGP, SHK, EFR, MF, and HHH conceived and designed research. JB, AGP, SHK, SP, and DO performed experiments. JB, EAR, AGP, SHK, SP, DO, CGS, MF, and HHH analyzed data. JB, EAR, AGP, SHK, SP, EFR, DO, CGS, RWO, US, MF, and HHH interpreted the results of the experiments. JB, EAR, AGP, and HHH prepared figures. JB, AGP, and HHH drafted the manuscript. JB, AGP, SHK, EFR, SP, US, MF, and HHH edited and revised the manuscript. All authors approved the final version of the manuscript.

## FUNDING INFORMATION

This work was supported by research grants from Innovation Fund Denmark (JB, #2050‐00018B; AGP, #1045‐00009B).

## CONFLICT OF INTEREST STATEMENT

JB, EAR, AGP, SHK, SP, DO, CGS, RWO, MF and HHH are employed by Gubra A/S. AGP, SP, DO, CGS, MF and HHH are shareholders in Gubra. EFR is employed by National Jewish Health. US is employed by Aarhus University, Aarhus, Denmark, and is a consultant and shareholder in Initiator Pharma A/S, which focuses on diseases unrelated to the current study.

## Data Availability

The RNA sequencing datasets generated in the current study are available in the Gene Expression Omnibus (GEO) repository (https://www.ncbi.nlm.nih.gov/geo/) with accession number GSE301216.
